# A comparison of indoor and outdoor malaria vectors in a longitudinal cohort study in Webuye, Western Kenya

**DOI:** 10.21203/rs.3.rs-10820457/v1

**Published:** 2026-09-08

**Authors:** Lucy Abel, Samuel Kahindi, Daniel Onguru, Shehu Awandu, Mark Amunga, Millicent Cherono, Linda Maraga, Dancan M. Wakoli, Evans Omollo, Erick Nalianya, Aggrey Wekesa, Christine Markwalter, Steve M. Taylor, Judith Mangeni, Andrew Obala, Wendy Prudhomme O’Meara

**Affiliations:** Academic Model Providing Access to Health Care (AMPATH), Kenya; Pwani University; Jaramogi Oginga Odinga University of Science and Technology; Jaramogi Oginga Odinga University of Science and Technology; Academic Model Providing Access to Health Care (AMPATH), Kenya; Academic Model Providing Access to Health Care (AMPATH), Kenya; Academic Model Providing Access to Health Care (AMPATH), Kenya; Academic Model Providing Access to Health Care (AMPATH), Kenya; Duke Global Inc; Duke Global Inc; Duke Global Inc; Duke University NC; Duke University NC; Moi University College of Health Sciences; Moi University College of Health Science; Duke University NC

**Keywords:** Indoor, outdoor, Anopheles

## Abstract

**Background:**

Malaria remains a major public health challenge in Kenya, with ~ 75% of the population is at risk. Despite the decline in malaria prevalence in children associated with sustained coverage of Insecticide Treated Nets in Kenya, this gain has not been uniform and there is a great deal of heterogeneity in transmission including areas with much higher transmission than the national average. This calls for a closer look at vector control to understand where it is failing. The main malaria vectors in Webuye are *Anopheles gambiae s.s, An. arabiensis* and *An. funestus* which are all known to be anthropophagic and endophagic. However, *An. arabiensis* has in recent times been reported to shift towards exophagic and zoophillic behavior which has been implicated in maintaining residual transmission. Despite scale-up of LLINs with PBO in the region, malaria burden remains high. Among the factors potentially sustaining transmission may be increased exophilic and exophagic vectors, thus reducing the efficacy of LLINs. We compared indoor and outdoor malaria vector diversity, *P. falciparum* infection, and measured the blood meal sources for the outdoor vectors.

**Methods:**

For 12 months, collection of adult *Anopheles* mosquitoes by aspiration was done weekly in buildings with human sleeping spaces (indoor), non-human dwellings and suspected mosquito resting areas (outdoor). Mosquitoes were identified morphologically and laboratory analysis was conducted by PCR.

**Results:**

Out of the 46,502 female mosquitoes collected,1,878 (4%) were anophelines. A total of 1,641 (87%) of the anophelines were collected indoors and 237 (13%) outdoors. The highest proportion of mosquitoes were collected between April and June. Ten different *Anopheles* species were identified indoors while six were identified outdoors. *Anopheles gambiae ss* was the most abundant vector both indoors and outdoors. A high proportion of the outdoor female *Anopheles* had bloodmeals from animals, only 3% fed on humans and all were negative for *Plasmodium* sporozoites. The sporozoite rate for indoor collection was 4.8%.

**Conclusion:**

There was no evidence that outdoor resting mosquitoes are contributing to malaria transmission. However, the presence of several other species besides the traditional vectors poses a threat of cryptic species sustaining transmission and the ability to feed on animals implicated in sustaining vector population.

## Introduction

Malaria remains a major a public health burden in sub-Saharan Africa. Despite the decline in malaria prevalence in children that is associated with sustained coverage of Insecticide Treated Nets in Kenya [1], it is observed that this gain has not been uniform and there is a great deal of heterogeneity in transmission including areas with much higher transmission than the national average even though ITN coverage resembles the national average. This calls for a closer look at vector control and prevention to understand where vector control is falling short.

The two main malaria vector control interventions in use in Kenya are the indoor residual spraying of insecticides (IRS) and the universal mass distribution of long-lasting insecticidal nets (LLINs). These two types of vector control measures exploit the malaria vector behaviour of biting humans indoors and late when people are deep asleep and rest indoors afterwards. However, the effectiveness of these two interventions can be greatly compromised by factors such as changes in vector biting behaviour [2, 3] and increased tolerance towards the insecticides used in the interventions by the vectors[4-7] .

*Anopheles* mosquitoes, which are known vectors for malaria, primarily feed on the blood of vertebrates [8]. Many *Anopheles* mosquitoes are highly anthropophilic, especially in areas where malaria is endemic [9]. They can also feed on the blood of various mammals, including cattle, sheep, goats, dogs, and cats, although the extent of non-human feeding varies by mosquito species. Some species of *Anopheles* may also take blood meals from birds, although this is less common compared to mammals[10]. The feeding behavior of *Anopheles* mosquitoes on animals besides humans has several implications for public health and vector control strategies. If *Anopheles* mosquitoes have alternative blood sources, it may reduce the effectiveness of vector control measures that target human-vector contact [11]. Furthermore, mosquitoes that can feed on animals may continue to thrive even in areas where human populations are protected [12].

Feeding behaviors such as choice of host, timing or location of foraging, are partly driven by the availability of hosts which varies based on environmental factors such as habitat, season, and the presence of humans or livestock [13, 14] as well as host availability due to the use of ITNs. Changes in these factors, including availability of hosts, may lead to behavioral adaptations, such as changes in feeding times or locations, which can make them harder to control [15]. Understanding the feeding behavior of *Anopheles* mosquitoes is crucial to informing public health strategies [16]. The ever changing vector feeding and resting behavior calls for an integrated approach to reduce malaria transmission [17] .

The main malaria vectors in western Kenya are *Anopheles gambiae ss, An. arabiensis* and *An. funestus ss*[18]. All the three vector species are primarily known to be anthropophagic and endophagic, however *An. arabiensis* has in recent times been reported to shift towards exophagy and preferring mammalian hosts other than humans [19]. This behaviour of biting and resting outdoors together with sourcing blood meals from diverse hosts has been implicated in maintaining residual malaria transmission [20].

The major vector control intervention in Bungoma County is LLINs which only targets indoor feeding and resting malaria vectors. Despite scale up and sustained coverage with LLINs through universal mass distributions, malaria transmission is still moderate and perennial with two seasonal peaks after long rains (May-June) and short rains (Sept-October) [21]. It is possible that persistent transmission is at least partially attributable to changes in vector behaviour such as host diversification, timing of biting, and possibly resting outdoors. Thus, this study aims to describe the biting behavior and sources of blood meal for the primary malaria vectors in Webuye, Bungoma County, and provide crucial insights on whether the current vector control measures (ITNs and IRS) continues to offer protection against malaria and further underpin where gaps in protection exist. To do so, we compare species composition, feeding success, bloodmeal source and sporozoite infection between indoor and outdoor collected mosquitoes from the same households over a one-year period. We hypothesized that the primary malaria vectors in Webuye could be shifting their bloodmeal seeking behavior towards hosts other than humans and also biting and resting outdoors thus contributing to residual malaria transmission.

## Materials and Methods

### Study Site

This study was conducted in four villages spread across Webuye East and Webuye West Sub Counties in Bungoma County, western Kenya, which is approximately 400 kilometres Northwest of Nairobi and 60 kilometres from Uganda border. The villages are rural and most families engage in small scale farming and livestock keeping. Sixty percent of families live below the poverty line and few have electricity or municipal water. *Plasmodium falciparum* transmission is perennial with seasonal peaks following the long rains in May-June and short rains in September-October. Ninety percent of homes in the study area have at least one insecticide-treated net, yet prevalence of *P. falciparum* in children less than 10 years of age exceeds 50% during the rainy season [21].

### Study Design

This study was conducted within an ongoing study in an existing longitudinal cohort design which was established in 2017 in Webuye, Bungoma County [21]. It builds on an ongoing work in the same cohort. This cohort was assembled in four villages using radial sampling of 20 households per village in three villages and 15 households in the fourth village beginning with a randomly selected household in each village. The four villages are spread across two sub-counties, Webuye East and West with a total of 75 enrolled households in all the villages. The existing cohort has households enrolled in clusters of about 0.25 kilometers radius [18]. The household population ranges from five to ten individuals per households and the total population for all the households is approximately 500 people.

### Field Data Collection

Mosquito collection was conducted weekly between February 2024 to January 2025 by use of prokopack aspiration. This is a highly maneuverable vacuum aspirator attached to an extension pole that can sample from the ground level up to 13 feet (4m) which enables mosquito collection to be made on upper walls, ceiling and under the furniture. The prokopack uses a 12 volt 12 amp hr gelled-electrolyte battery with plastic collection cups made with stainless steel mosquito mesh at the bottom [22]. This mosquito collection was selected due to its cost effectiveness, convenience and the ability to collect both indoor and outdoor resting mosquitoes. The collection was done early morning between 6.00 to 8.30am. The participants were asked to leave the doors and windows closed until the team arrived. Collections of resting adult *Anopheles* mosquitoes was made from both indoor and outdoor mosquito resting areas. One collection cup was used per building and one for outdoor collections and labelled by collection date and point. The indoor mosquito resting areas includes the walls, ceiling and underneath the furniture in the household including the sleeping spaces while the outdoor mosquito resting areas include pit latrines, animal shelters, outside kitchens and other buildings or structures within the compound that are not used as human sleeping spaces [22-24].

### Laboratory processing of mosquito samples

The mosquito samples were transported to the laboratory in a cool box with an ice pack for sorting and grading of the abdominal status. *Anopheles* mosquitoes were identified to species using morphological features [25] .

### DNA Extraction

Wings of the indoor resting mosquitoes and the abdomens of the outdoor resting mosquitoes were extracted by Hotshot DNA extraction method as described by Truett [26]. Briefly, the samples were individually placed into wells of a 96-well plate. Fifty microliters of alkaline lysis buffer were added to each well containing the mosquito sample, and the plate was incubated at 95°C for 30 minutes. An equal volume of neutralizing solution (Tris HCl pH 5.0) was added, and plates were subsequently stored at −20°C.

The thoraces of the mosquitoes were extracted using the Chelex extraction method [27]. Briefly the mosquito thoraces were homogenized and 75μL of nuclease-free water and 25μL of 20% Chelex was added to each tube with the sample. The mosquito pellets were re-suspended in the chelex solution with gentle vortexing and spin down for 2 seconds then the tubes incubated in 99°C water bath for 12 minutes with vortexing at 3 minutes interval. After incubation the tubes were cooled for 3 minutes and spun at 12,000xg for 3min. The supernatant containing sporozoite DNA from each tune were transferred into a new microcentrifuge tube leaving the pellets and the chelex beads undisturbed and this was tested with PCR.

### Species identification by PCR

Morphologically indistinguishable members of *Anopheles gambiae* complex were identified further by Polymerase Chain Reaction (PCR) to distinguish *An. gambiae s.s*, and *An.arabiensi*s. Multiplex PCR was run on 5 μl of hotshot extracted indoor wings and outdoor abdomens following the method of Wilkins et al [28] Reactions were run on a 1% agarose gel at 100V for 40mins. DNA fragments were visualized under UV light using SYBR safe stain added to the agarose gel. The *An. gambie sl* that did not amplify as either *An. gambiae ss* or *An. arabiensis* maintained their species identification as *An. gambiae sl.*

### Host blood meal analysis

Identification of the host blood meal for outdoor door resting *Anopheles* species was done using conventional PCR on the extracted abdomens. All the anopheles mosquitoes collected outdoors regardless of their visible abdominal status were tested for the presence of human or animal bloodmeal [29]. The PCR amplifies the 12S ribosomal RNA gene with a product size of 205bp for any vertebrate DNA. Another product of 154bp for human blood meal is also amplified if human genomic DNA is present. The PCR product was then run on a 2% agarose gel.

#### Plasmodium falciparum detection

To measure the sporozoite rate of outdoor resting *Anopheles* mosquitoes and compare the sporozoite rate of indoor resting *Anopheles* mosquitoes, the thoraces of *Anopheles* mosquitoes collected both indoor and outdoor were tested for the presence of *P. falciparum* genomic DNA using Realtime quantitative PCR (RT-qPCR). The RT-qPCR assay that was used targets a multi-copy motif in the *P. falciparum* genome called pfr364 [30] which can detect parasites down to as low as 1 x 10 − 6 ng/uL of parasite genomic DNA.

### Data analysis

Data were entered into Redcap and laboratory results were compiled in an excel spreadsheet and both were merged for data analysis. The descriptive statistics were supplemented with Fisher’s Exact tests to compare sporozoite and bloodmeal source across Female Anopheles species. The human blood index (HBI) was calculated as the total number of mosquito fed on human/ total number of female *Anopheles* mosquitoes tested regardless of their abdominal status. Sporozoite rate was calculated as (number of positive mosquitoes /numbers of analyzed mosquitoes)*100. Data analysis and visualization was done on RStudio Version 2025.09.1 + 401 based on RV4.5.1

## Results

### General mosquito Indoor and Outdoor distribution

Mosquito collection was conducted weekly per household between February 2024 to January 2025 in 75 households for a total of 36 nights of collection per household. Overall, 46502 female mosquitoes were collected both indoors (n = 38713, 83%) and outdoors (n = 7789, 17%). Out of these only 1878 (4%) were anophelines and 44624 (96%) were culicines. About 1641 (87%) of the female anophelines were collected indoors while 237 (13%) were collected outdoors. There is sufficient evidence to conclude that the distribution of mosquitoes between indoor and outdoor collection sites differs by genus. Specifically, *Anopheles* mosquitoes were proportionally more likely to be collected indoors than *Culex* mosquitoes (Table 1).

A total of 10 different *Anopheles* species were recorded indoors while 6 were recorded outdoors. *Anopheles gambiae ss* was the most abundant species found indoors (n = 915, 55%) followed by *An. funestus* (n = 529, 32%), *An. gambiae sl* (n = 71, 4%), *An. arabiensis* (n = 50, 3%) and *An. demeilloni* (n = 47, 2.8%) (Fig. 1a). *An. gambiae ss* was also the most abundant species collected outdoors (n = 84, 36%), followed by *An. gambiae sl* (n = 67, 28%) *An. funestus* (n = 52, 22%), *An. demeilloni* (n = 17, 7%) *An. arabiensis* (n = 10,4%) and *An. rufipes* (n = 7, 3%) (Fig. 1b). Overall, there was greater diversity of species in the indoor collections but the proportion of different species was more evenly distributed in outdoor collections.

Overall, Maruti village had the highest number of female *Anopheles* mosquitoes collected from both indoors and outdoors while Sitabicha village had the lowest. There were geographic heterogeneities in species distribution. Among indoor mosquitoes, *Anopheles funestus* was more prevalent in Maruti and Kinesamo and roughly equal to *An. gambaie s.s.* while *An. gambiae s.s.* dominated in Nangili and Sitabicha. A similar pattern was seen in outdoor collections, except that the proportion of *An. gambiae s.l*. (identified morphologically) that could not be resolved to either *An. gambiae s.s.* or *An. arabiensis* by PCR was much larger in outdoor collections across all villages. (Fig. 2a and 2b)

### Monthly trends of indoor and outdoor female Anopheles mosquitoes

The highest proportion of mosquitoes were collected in the period between April and June for both indoor and outdoor collections (Fig. 3a and 3b) after which there was a steady decline for the remaining months. The various species of female anophelines were fairly distributed across all the collection months. However, during the peak period, *An. gambiae* complex comprised the highest proportion for indoor collections followed by *An. funestus*.

#### Source of blood meal in female Anopheles mosquitoes

Blood meal analysis was completed for 234 out of the 238 female *Anopheles* mosquitoes that were collected outdoors to determine whether outdoor resting mosquitoes were feeding on human or non-human sources. A total of 7 mosquitoes had blood meal from humans while 138 mosquitoes had fed on other mammals (Table 2). With regard to species, only *An. funestus* and *An. gambiae ss* had human blood meals at ratios of 5.9% and 4.8% respectively. *Anopheles arabiensis* had the highest ratio of blood meal from other mammals followed by *An. gambiae ss. An. gambaie s.l.* had the highest proportion unfed.

The human blood index (HBI) was 0.03. There was a statistically significant association between female *Anopheles* species and their source of blood meal (Fisher’s Exact Test, *p* = 0.0001) (Fig. 4) while the association between the blood meal source across the villages was not statistically significant (Fisher’s Exact Test, p = 0.0699) (Fig. 5).

#### Sporozoite rates for in Indoor and Outdoor Anopheles mosquitoes

A total of 1846 female *Anopheles* mosquitoes were tested for *P. falciparum* genomic DNA (1612 indoor and 234 outdoor) with 4.2% turning positive. *P. falciparum* genomic DNA was detected in 4.8% of the female *Anopheles* species collected indoors (Table 3) and none in all the outdoor collected female *Anopheles* mosquitoes. *Anopheles gambiae sl* had the highest *Plasmodium* infection rate at 7% followed by *An. gambiae s.s* (5%), *An. funestus* (4.8%) and *An. demeilloni* (4.5%) (Table 3).

Nangili village recorded the highest *Plasmodium* sporozoites rates in *Anopheles* mosquitoes at 6% and followed closely by Maruti and Kinesamo villages at 5.1% and 4.7% respectively. Sitabicha had the lowest sporozoite rate at 1.2%. The association between the *Anopheles* species and the *P. falciparum* infection rate was not statistically significant (Fisher’s Exact Test, p = 0.102) (Fig. 6).

## Discussion

Outdoor malaria transmission is hypothesized to be a significant threat to malaria control especially where mosquitoes bite people outdoors while the interventions used are indoor based. Furthermore, it has been suggested that the pressure exerted by chemical based interventions has contributed to pushing Anopheles mosquitoes that were mainly endophilic to shift towards resting outdoors. Mosquitoes likely select resting sites with suitable microclimatic conditions in order to minimize physiological stress during the resting phase [31].

*Anopheles gambiae* complex remains the dominant vector species collected, and this is consistent with other studies done in western Kenya [32, 33]. Fewer mosquitoes were found in outdoor non-dwelling structures compared to indoors. Overall, the diversity of these two populations was similar although the distribution of species was different. Some vectors that are typically thought of as dedicated endophilic were collected from outdoor resting places, such as *An.gambiae ss* and *An. funestus*. A larger proportion of *An. funestus* were found in the indoor collection, while a substantial proportion of the outdoor collection were *An gambiae* complex that were not identified to *An gambiae ss*, *An. arabiensis* or *An. colluzzi* by PCR. A study on outdoor malaria vector species in dryland ecosystems of Kenya similarly reported the presence of cryptic species [34]. The presence of several other species besides the common vectors, although negative for sporozoites, pose a threat of secondary vectors sustaining transmission [35]. Unlike the secondary vector species identified here, the unidentified *An gambiae* complex members had higher infection rates than any other group and raise the possibility of transmission being fueled by cryptic or uncharacterized vector species. Overall, this suggest that outdoor resting mosquitoes are not simply a subset of the indoor resting population that have modified their behavior, nor are they an entirely different set of species, but potentially some combination of the two.

A substantial proportion of the outdoor mosquitoes had fed, with feeding rates among *An gambiae ss* as high as the indoor population [21]. Surprisingly, nearly three quarters had fed on non-human blood sources compared to 69% fed on human sources from indoor collections [36]. Non-human bloodmeals have been detected in *An. gambaie s.s.* previously and this is a strong signal of behavior adaptation possibly due to the high coverage with LLINs[37]. Although most outdoor mosquitoes had animal bloodmeals, meaning they likely fed outside, a few human bloodmeals were found in vectors known to be highly anthropophilic. It is not known if they fed outdoors or fed indoors then exited to rest. Either of these represents a behavioral adaptation although each would require different mitigation strategies.

Although the vectors tested in this study had moderately high *P. falciparum* infection rates, no outdoor mosquitoes had sporozoites and this has also been reported in other studies [38], so they cannot be definitively implicated in malaria transmission. Other studies have similarly documented low to no *P.falciparum* infection in outdoor collected mosquitoes [39]. However, given the human bloodmeals identified in outdoor resting mosquitoes, it is plausible that they could contribute to transmission and a larger study would be needed to definitively answer this question. In addition, outdoor resting mosquitoes have been reported to show some levels of pyrethroid resistance [40] which could compound the challenge to malaria interventions.

### Limitations

Since this study was nested in a larger study, we were unable to test the bloodmeal sources of the indoor collected which were already used for a separate investigation. In addition, we were not able to expand our molecular testing to include other sibling species of *An.gambiae sl* besides the most common *An.arabiensis*, *An.gambiae ss, and An. coluzzi* (of which there were none) therefore the mosquitoes that did not amplify for these species remained grouped as *An.gambiae sl* although they almost certainly do not represent a single homogenous group.

## Conclusion

The study demonstrated high species diversity of malaria vectors in Webuye, Kenya resting both indoors and outdoors, with the main malaria vectors species being *An. gambiae s.s. An. arabiensis* and *An. funestus*. Traditionally, *An. gambiae s.s* and *An. funestus* are known to be endophagic and endophilic, however in this study they have shown a capacity to feed or rest outdoors. Furthermore, some of the vectors caught outdoors had human blood meals and although none was sporozoite positive, this raises the potential of outdoor malaria transmission which is out of target of the current vector interventions; the LLINs and IRS. Better understanding of where people are being bitten is needed in order to design effective interventions that could reduce outdoor transmission.

## Figures and Tables

**Figure 1.**
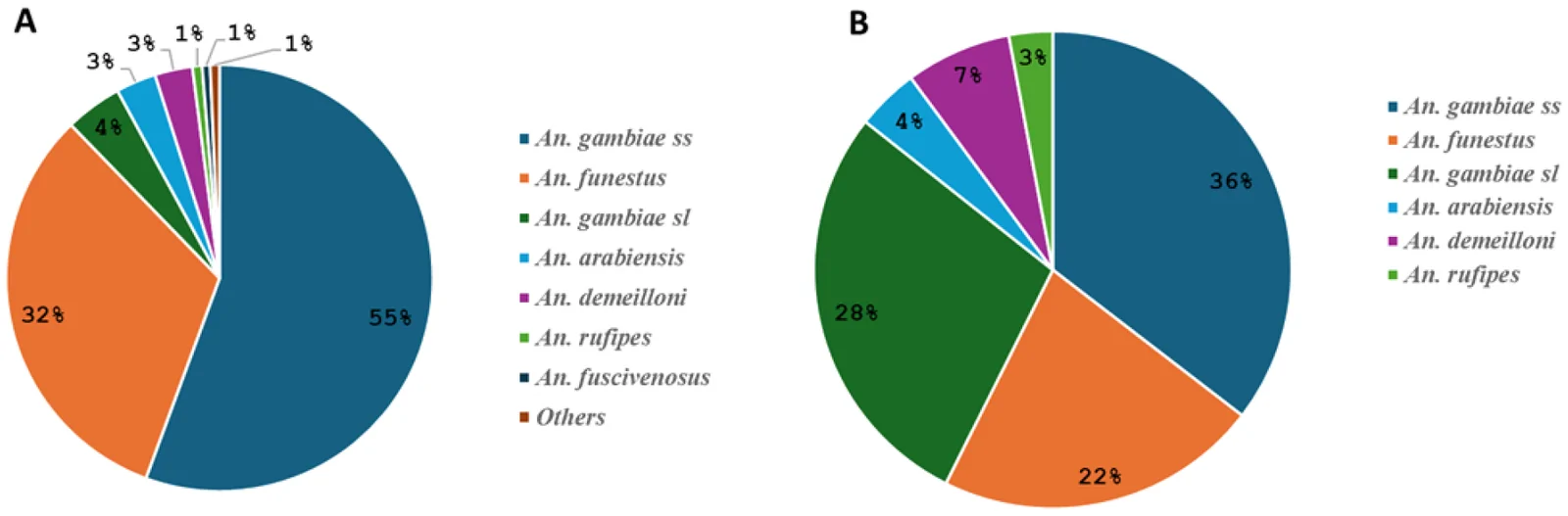
**(A)** Distribution of Indoor Female *Anopheles* species Key: Others= *An. coustani 3, An. garnhani 1, An. pretoriensis* 1 and undetermined 2. **(B)** Distribution of Outdoor Female *Anopheles* species

**Figure 2.**
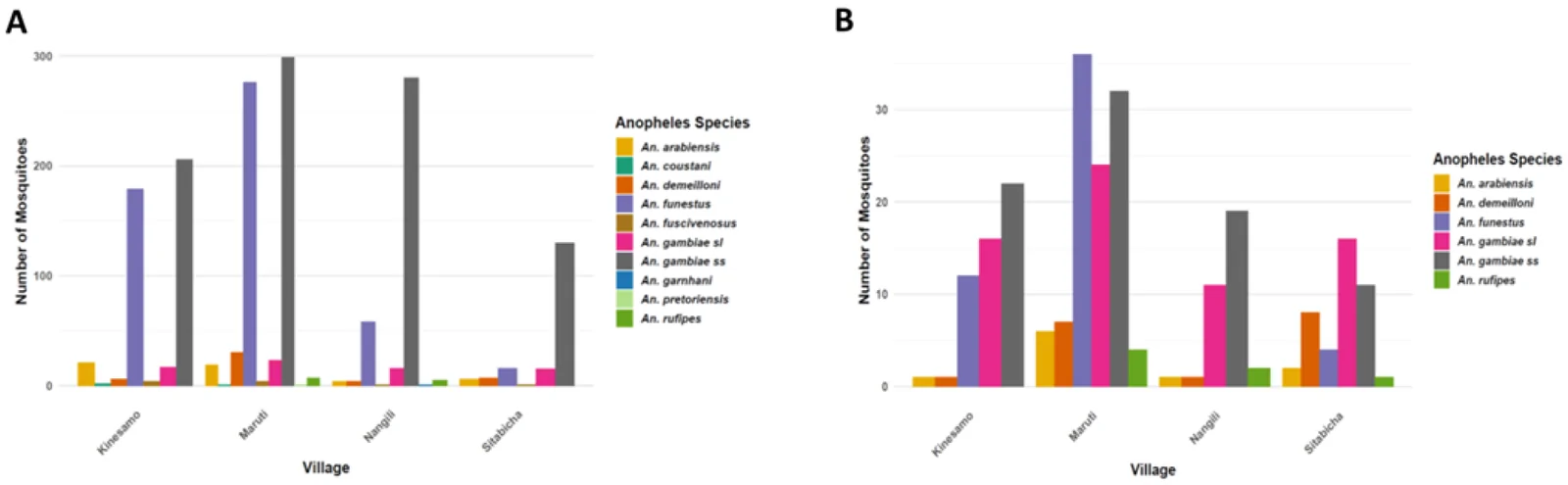
**(A)** Distribution of Indoor Female *Anopheles* species per village **(B)** Distribution of Outdoor Female *Anopheles* species per village

**Figure 3.**
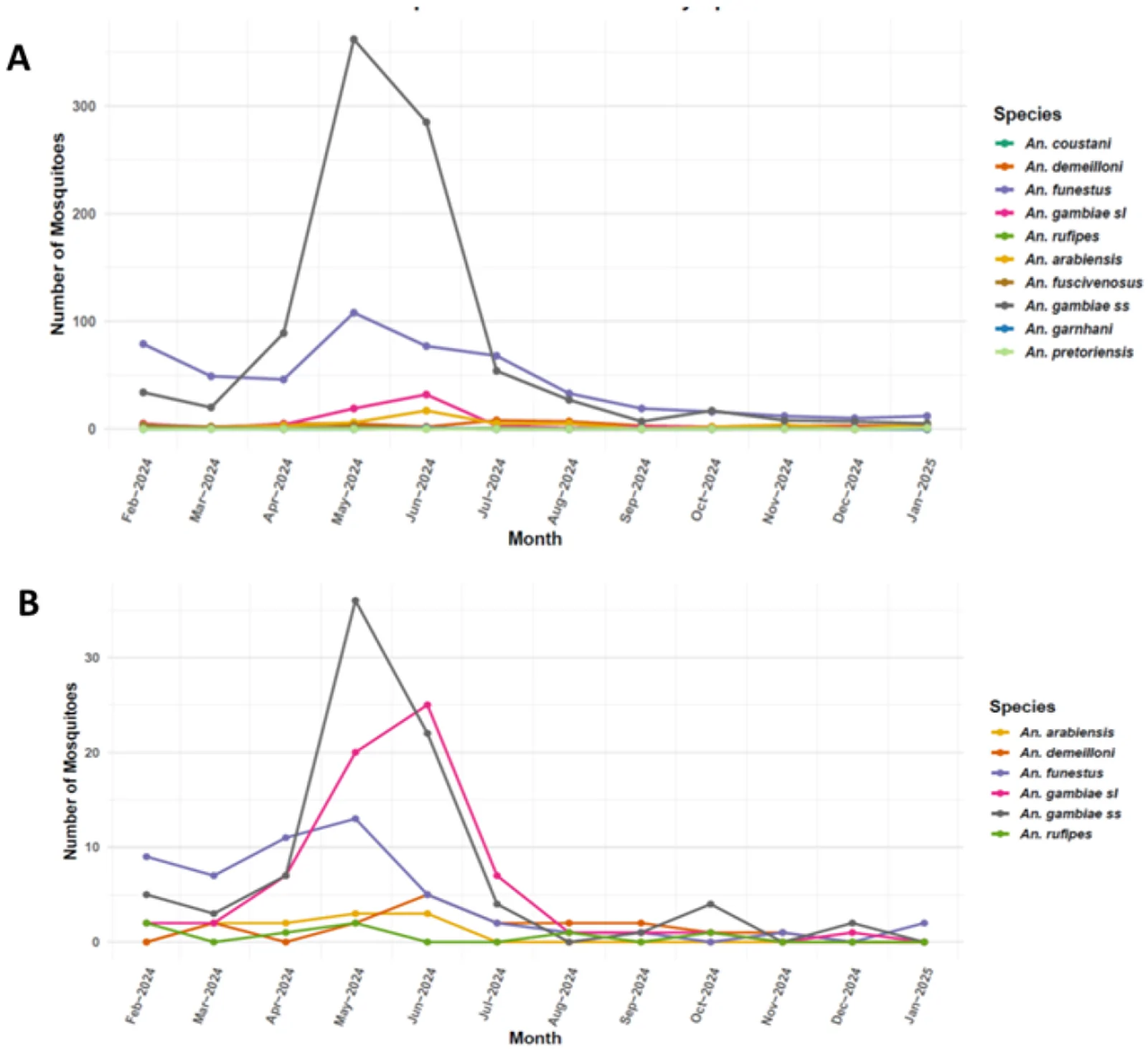
**(A)** Trend of monthly indoor female *Anopheles* mosquitoes **(B)** Trend of monthly outdoor female *Anopheles* mosquitoes

**Figure 4.**
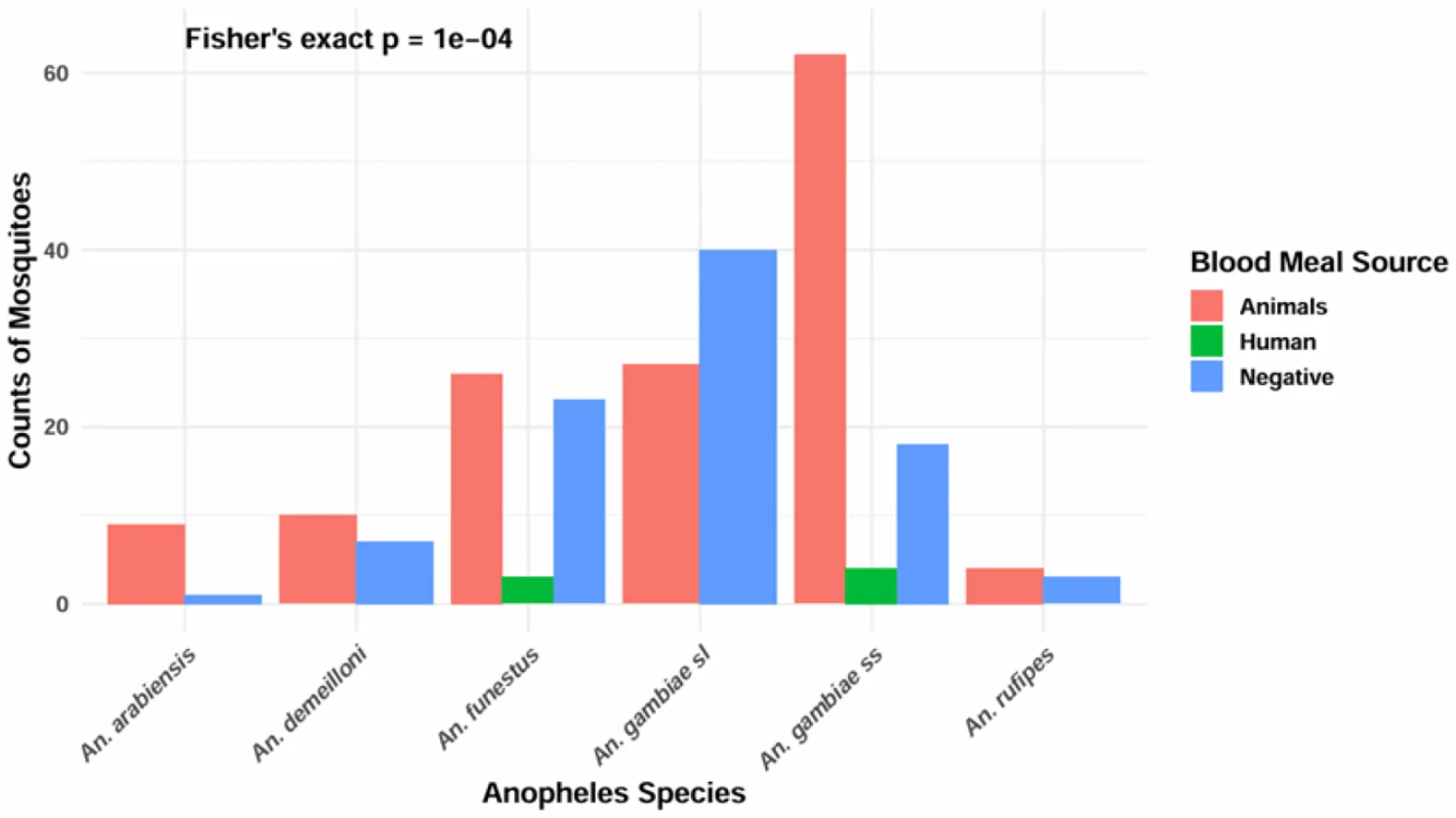
Blood meal sources for outdoor female *Anopheles* species

**Figure 5.**
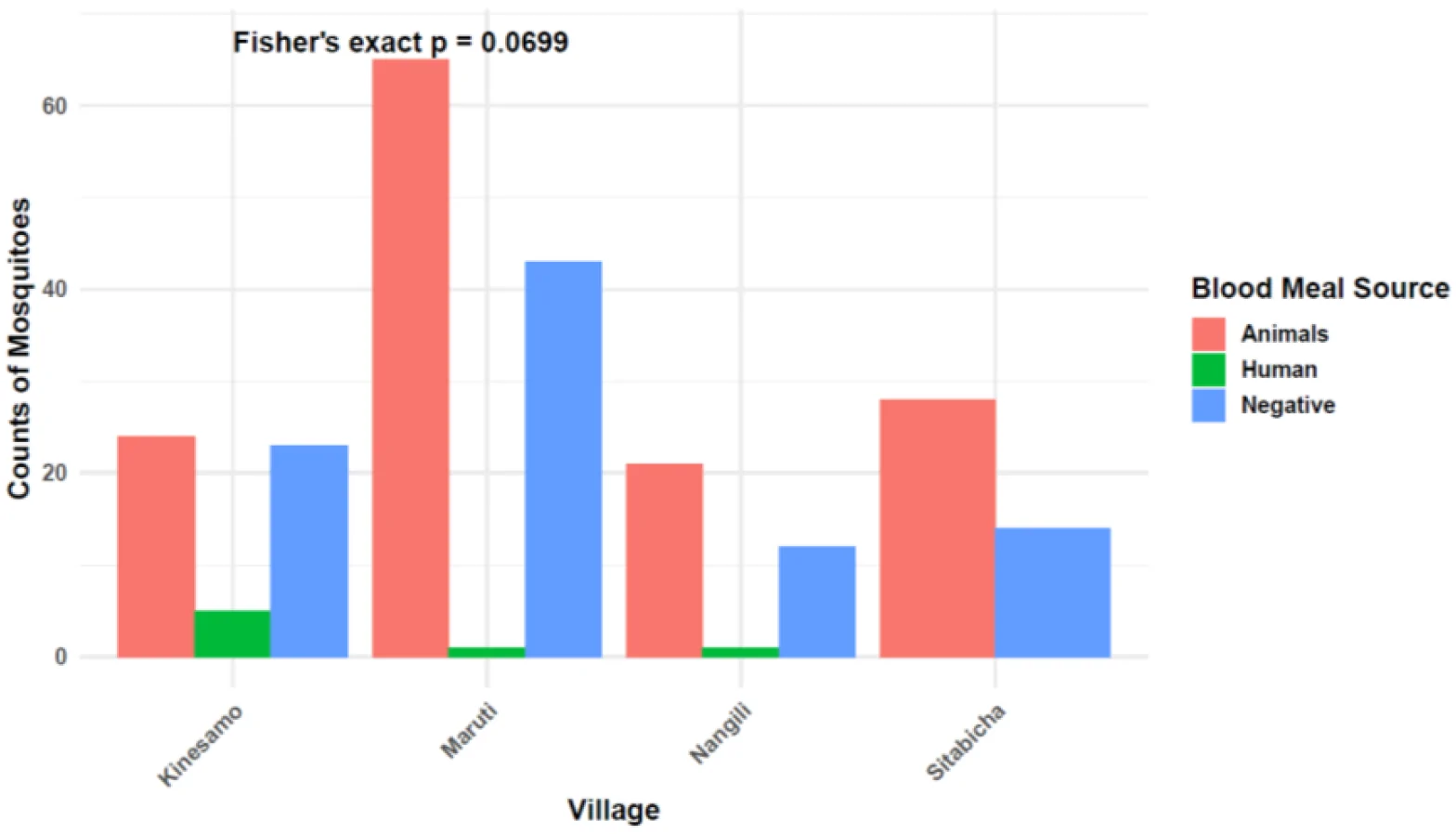
Blood meal sources by Village

**Figure 6.**
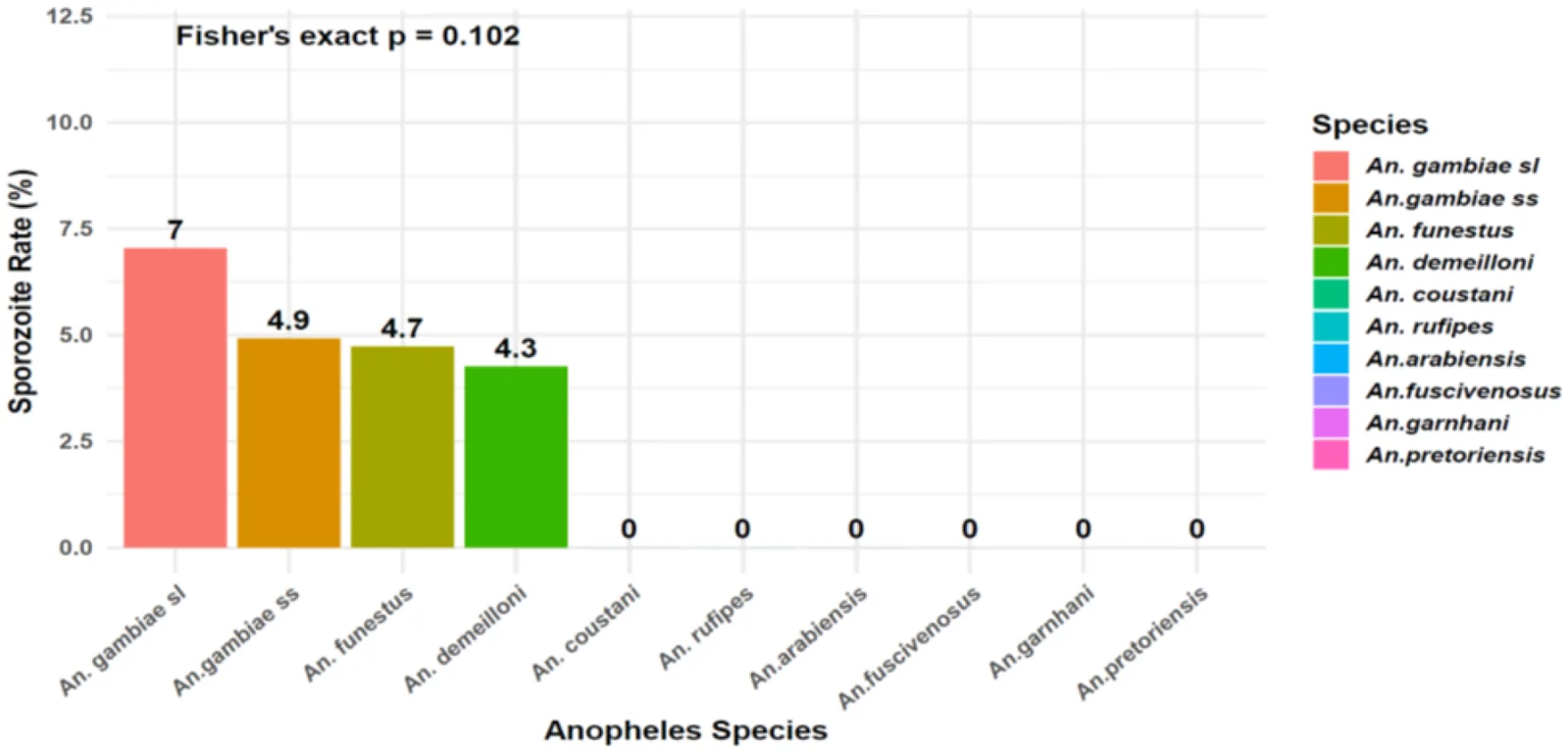
Distribution of *Plasmodium* sporozoites in the Indoor *Anopheles* species

**Table 1:** Mosquito distribution per Genus and collection site

Mosquito Genus	Collection site		Total
	Indoor	Outdoor	
*Anopheles*	1641 (87.4%)	237 (12.6%)	1878 (4%)
*Culex*	37072 (83.1%)	7552 (16.9%)	44624 (96%)
Total	38713 (83.2%)	7789 (16.8%)	46502 (100%)
**Chi-square test**	**χ^2^ = 23.89**	**df = 1**	**p < 0.05**

**Table 2:** Distribution of Blood meals sources in the Outdoor *Anopheles* species

Species	Human positive	Blood meal negative	Other mammals	Undetermined	Total
*An. arabiensis*	0 (0.0%)	1 (10%)	9 (90.0%)	0 (0.0%)	10
*An. demeilloni*	0 (0.0%)	7 (41.2%)	10 (58.8%)	0 (0.0%)	17
*An. funestus*	3 (5.9%)	22 (43.1%)	26 (51.0%)	0 (0.0%)	51
*An. gambiae sl*	0 (0.0%)	37 (56.9%)	27 (41.5%)	1 (1.5%)	65
*An. gambiae ss*	4 (4.8%)	18 (21.4%)	62 (73.8%)	0 (0.0%)	84
*An. rufipes*	0 (0.0%)	3 (42.9%)	4 (57.1%)	0 (0.0%)	7
**Total**	**7**	**88**	**138**	**1**	**234**

**Table 3:** Distribution of *Plasmodium* sporozoites in the Indoor *Anopheles* species

Species	Sporozoite −ve	Sporozoite +ve	Total
*An. coustani*	3 (100%)	0 (0%)	3
*An. demeilloni*	42 (95.5%)	2 (4.5%)	44
*An. funestus*	492 (95.2%)	25 (4.8%)	517
*An. gambiae sl*	66 (93%)	5 (7%)	71
*An. rufipes*	11 (100%)	0 (0.0%)	11
*An.arabiensis*	47 (100%)	0 (0.0%)	47
*An.fuscivenosus*	10 (100%)	0 (0.0%)	10
*An.gambiae ss*	860 (95%)	45 (5%)	905
*An.garnhani*	1 (100%)	0 (0.0%)	1
*An.pretoriensis*	1 (100%)	0 (0.0%)	1
*Undetermined*	1 (50%)	1 (50%)	2
**Total**	**1534**	**78**	**1612**

## Data Availability

The datasets used and/or analyzed during the current study are freely available upon request.

## List of abbreviations

*An*: *Anopheles*
DDT: Dichlorodiphenyltrichloroethane
DNA: Deoxyribonucleic acid
HBI: Human Blood Index
IRB: Institutional Review Board
IREC: Institutional Review and Ethics Committee
IRS: Indoor Residual Spraying
ITNs: Insecticide Treated Nets
LSM: Larval Source Management
LLINs: Long Lasting Insecticidal Nets
NACOSTI: National Commission for Science, Technology & Innovation
MoH: Ministry of Health
*P. falciparum*: *Plasmodium falciparum*
PCR: Polymerase Chain Reaction
PEARL: Partnership for Education and Research Laboratory
RNA: Ribonucleic acid
WHO: World Health Organization
